# Incidence and Trends of Infections with Pathogens Transmitted Commonly Through Food and the Effect of Increasing Use of Culture-Independent Diagnostic Tests on Surveillance — Foodborne Diseases Active Surveillance Network, 10 U.S. Sites, 2013–2016

**DOI:** 10.15585/mmwr.mm6615a1

**Published:** 2017-04-21

**Authors:** Ellyn P. Marder, Paul R. Cieslak, Alicia B. Cronquist, John Dunn, Sarah Lathrop, Therese Rabatsky-Ehr, Patricia Ryan, Kirk Smith, Melissa Tobin-D’Angelo, Duc J. Vugia, Shelley Zansky, Kristin G. Holt, Beverly J. Wolpert, Michael Lynch, Robert Tauxe, Aimee L. Geissler

**Affiliations:** ^1^Division of Foodborne, Waterborne, and Environmental Diseases, National Center for Emerging and Zoonotic Infectious Diseases, CDC; ^2^Oregon Health Authority; ^3^Colorado Department of Public Health and Environment; ^4^Tennessee Department of Health; ^5^University of New Mexico; ^6^Connecticut Department of Public Health; ^7^Maryland Department of Health and Mental Hygiene; ^8^Minnesota Department of Health; ^9^Georgia Department of Public Health; ^10^California Department of Public Health; ^11^New York State Department of Health; ^12^Food Safety and Inspection Service, U.S. Department of Agriculture, Atlanta, Georgia; ^13^Center for Food Safety and Applied Nutrition, Food and Drug Administration, Silver Spring, Maryland.

Foodborne diseases represent a substantial public health concern in the United States. CDC’s Foodborne Diseases Active Surveillance Network (FoodNet) monitors cases reported from 10 U.S. sites* of laboratory-diagnosed infections caused by nine enteric pathogens commonly transmitted through food. This report describes preliminary surveillance data for 2016 on the nine pathogens and changes in incidences compared with 2013–2015. In 2016, FoodNet identified 24,029 infections, 5,512 hospitalizations, and 98 deaths caused by these pathogens. The use of culture-independent diagnostic tests (CIDTs) by clinical laboratories to detect enteric pathogens has been steadily increasing since FoodNet began surveying clinical laboratories in 2010 (*1*). CIDTs complicate the interpretation of FoodNet surveillance data because pathogen detection could be affected by changes in health care provider behaviors or laboratory testing practices (*2*). Health care providers might be more likely to order CIDTs because these tests are quicker and easier to use than traditional culture methods, a circumstance that could increase pathogen detection (*3*). Similarly, pathogen detection could also be increasing as clinical laboratories adopt DNA-based syndromic panels, which include pathogens not often included in routine stool culture (*4*,*5*). In addition, CIDTs do not yield isolates, which public health officials rely on to distinguish pathogen subtypes, determine antimicrobial resistance, monitor trends, and detect outbreaks. To obtain isolates for infections identified by CIDTs, laboratories must perform reflex culture^†^; if clinical laboratories do not, the burden of culturing falls to state public health laboratories, which might not be able to absorb that burden as the adoption of these tests increases (*2*). Strategies are needed to preserve access to bacterial isolates for further characterization and to determine the effect of changing trends in testing practices on surveillance.

FoodNet is a collaboration among CDC, 10 state health departments, the U.S. Department of Agriculture’s Food Safety and Inspection Service, and the Food and Drug Administration. FoodNet personnel conduct active, population-based surveillance for laboratory-diagnosed infections caused by *Campylobacter*, *Cryptosporidium*, *Cyclospora*, *Listeria*, *Salmonella*, Shiga toxin-producing *Escherichia coli* (STEC), *Shigella*, *Vibrio*, and *Yersinia* in 10 sites covering approximately 15% of the U.S. population (an estimated 49 million persons in 2015). Confirmed bacterial infections are defined as isolation of the bacterium from a clinical specimen by culture. Confirmed parasitic infections are defined as detection of the parasite from a clinical specimen by direct fluorescent antibody test, polymerase chain reaction, enzyme immunoassay, or light microscopy. CIDTs detect bacterial pathogen antigen, nucleic acid sequences, or for STEC, Shiga toxin or Shiga toxin genes, in a stool specimen or enrichment broth.^§^ A CIDT positive–only bacterial infection is a positive CIDT result that was not confirmed by culture. Hospitalizations occurring within 7 days of specimen collection are recorded. The patient’s vital status at hospital discharge (or 7 days after specimen collection if not hospitalized) is also recorded. Hospitalizations and deaths occurring within 7 days of specimen collection are attributed to the infection. FoodNet also conducts surveillance for physician-diagnosed postdiarrheal hemolytic uremic syndrome (HUS), a potential complication of STEC infection, by review of hospital discharge data through a network of nephrologists and infection preventionists. This report includes HUS cases among persons aged <18 years for 2015, the most recent year with available data.

Incidence of infection for each pathogen is calculated by dividing the number of infections in 2016 by the U.S. Census estimates of the surveillance area population for 2015. Incidence is calculated for confirmed infections alone and for confirmed or CIDT positive–only infections combined. A negative binomial model with 95% confidence intervals (CIs) was used to estimate changes in incidence of confirmed bacterial and parasitic infections and confirmed or CIDT positive–only bacterial infections in 2016 compared with 2013–2015, adjusting for changes in the surveillance population over time. For STEC, incidence is reported for all STEC serogroups combined because it is not possible to distinguish between serogroups using CIDTs. Insufficient data were available to assess change for *Cyclospora*. For HUS, the 2015 incidence was compared with incidence during 2012–2014.

## Cases of Infection, Incidence, and Trends

During 2016, FoodNet identified 24,029 cases, 5,512 hospitalizations, and 98 deaths caused by confirmed or CIDT positive–only infections (Table 1). The largest number of confirmed or CIDT positive–only infections in 2016 was reported for *Campylobacter* (8,547), followed by *Salmonella* (8,172), *Shigella* (2,913), STEC (1,845),^¶^ *Cryptosporidium* (1,816), *Yersinia* (302), *Vibrio* (252), *Listeria* (127), and *Cyclospora* (55). The proportion of infections that were CIDT positive without culture confirmation in 2016 was largest for *Campylobacter* (32%) and *Yersinia* (32%), followed by STEC (24%), *Shigella* (23%), *Vibrio* (13%), and *Salmonella* (8%). The overall increase in CIDT positive–only infections for these six pathogens in 2016 was 114% (range = 85%–1,432%) compared with 2013–2015. Among infections with a positive CIDT result in 2016, a reflex culture was attempted on approximately 60% at either a clinical or state public health laboratory. The proportion of attempted reflex cultures differed by pathogen, ranging from 45% for *Campylobacter* to 86% for STEC and 88% for *Vibrio* (Figure). Among infections for which reflex culture was performed, the proportion of infections that were positive was highest for *Salmonella* (88%) and STEC (87%), followed by *Shigella* (64%), *Yersinia* (59%), *Campylobacter* (52%), and *Vibrio* (46%).

**TABLE 1:** Number of confirmed and CIDT positive–only* bacterial and confirmed parasitic infections, hospitalizations, and deaths, by pathogen — FoodNet, 10 U.S. sites,^†^ 2016^§^

Pathogen	Confirmed			Confirmed or CIDT positive–only		
	No. cases	Hospitalizations	Deaths	No. cases	Hospitalizations	Deaths
		No. (%)	No. (%)		No. (%)	No. (%)
**Bacteria**						
*Campylobacter*	5,782	1,082 (19)	10 (0.2)	8,547	1,697 (20)	26 (0.3)
*Listeria*^¶,^**	127	123 (97)	17 (13.4)	127	123 (97)	17 (13.4)
*Salmonella*	7,554	2,163 (29)	39 (0.5)	8,172	2,255 (28)	40 (0.5)
*Shigella*	2,256	519 (23)	2 (0.1)	2,913	579 (20)	2 (0.1)
STEC^††^	1,399	326 (23)	3 (0.2)	1,845	408 (22)	3 (0.2)
*Vibrio*	218	61 (28)	4 (1.8)	252	73 (29)	4 (1.6)
*Yersinia*	205	54 (27)	3 (1.5)	302	83 (28)	3 (1.0)
**Parasite**						
Cryptosporidium**	1,816	291 (16)	3 (0.2)	1,816	291 (16)	3 (0.2)
Cyclospora**	55	3 (5)	0 (—)	55	3 (5)	0 (—)
**Total**	**19,412**	**4,622**	**81**	**24,029**	**5,512**	**98**

**Abbreviations:** CIDT = culture-independent diagnostic test; FoodNet = CDC’s Foodborne Diseases Active Surveillance Network; STEC = Shiga toxin-producing *Escherichia coli*.

* CIDT positive–only is defined as detection of the bacterial pathogen, or for STEC, Shiga toxin, or the genes that encode a Shiga toxin, in a stool specimen or enrichment broth using a CIDT. Any positive CIDT result that was confirmed by culture is counted only among the confirmed infections. For STEC, only CIDT reports that were positive at a state public health laboratory were counted.

^†^ Connecticut, Georgia, Maryland, Minnesota, New Mexico, Oregon, Tennessee, and selected counties in California, Colorado, and New York.

^§‑^Data for 2016 are preliminary.

^¶^ *Listeria* cases are defined as isolation of *L. monocytogenes* from a normally sterile site or, in the setting of miscarriage or stillbirth, isolation of *L. monocytogenes* from placental or fetal tissue.

** All *Listeria*, *Cryptosporidium*, and *Cyclospora* infections were confirmed, so confirmed numbers are displayed in both columns.

^††^ For STEC, all serogroups were combined as it is not possible to distinguish between serogroups using CIDTs. Shiga toxin–positive reports from clinical laboratories that were Shiga toxin–negative at a state public health laboratory were excluded (n = 568).

**FIGURE:**
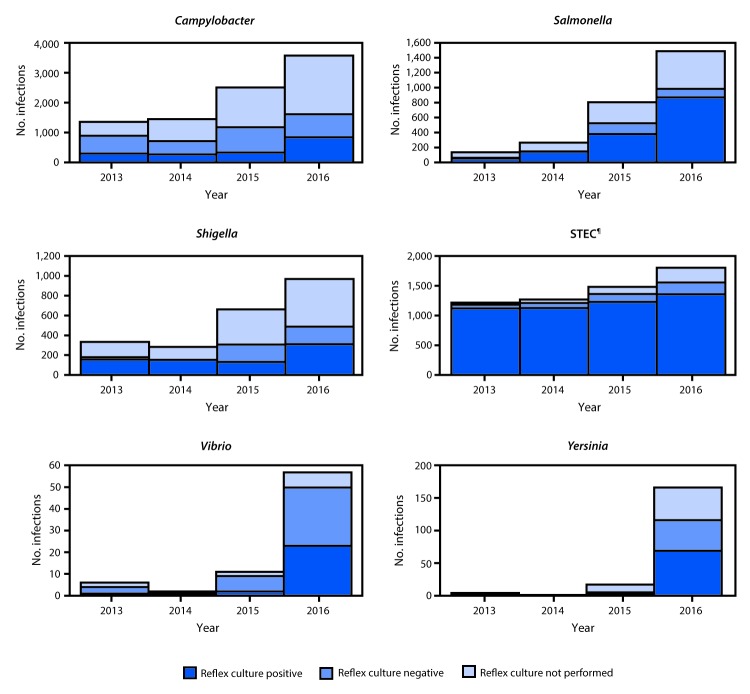
Number of infections with positive culture-independent diagnostic test (CIDT) results,* by pathogen, year, and culture status — FoodNet, 10 U.S. sites,^†^ 2013–2016^§^ **Abbreviations:** FoodNet = CDC’s Foodborne Diseases Active Surveillance Network; STEC = Shiga toxin–producing *Escherichia coli*. * Positive CIDT results are defined as detection of the bacterial pathogen, or for STEC, Shiga toxin or the genes that encode a Shiga toxin in a stool specimen or enrichment broth using a CIDT. For STEC, only CIDT results that were positive at a state public health laboratory were counted. ^†^ Connecticut, Georgia, Maryland, Minnesota, New Mexico, Oregon, Tennessee, and selected counties in California, Colorado, and New York. ^§^ Data for 2016 are preliminary. ^¶^ For STEC, all serogroups were combined because distinguishing between serogroups using CIDTs is not possible. Shiga toxin–positive reports from clinical laboratories that were Shiga toxin–negative at a state public health laboratory were excluded (n = 568).

The incidence of confirmed infections and of confirmed or CIDT positive–only infections per 100,000 persons was highest for *Campylobacter* (confirmed = 11.79; confirmed or CIDT positive–only = 17.43) and *Salmonella* (15.40; 16.66), followed by *Shigella* (4.60; 5.94), *Cryptosporidium* (3.64; N/A**), STEC (2.85; 3.76), *Yersinia* (0.42; 0.62), and lowest for *Vibrio* (0.45; 0.51), *Listeria* (0.26; N/A), and *Cyclospora* (0.11; N/A) (Table 2). Compared with 2013–2015, the 2016 incidence of *Campylobacter* infection was significantly lower (11% decrease) when including only confirmed infections, yet was not significantly different when including confirmed or CIDT positive–only infections. Incidence of STEC infection was significantly higher for confirmed infections (21% increase) and confirmed or CIDT positive–only infections (43% increase). Similarly, the incidence of *Yersinia* infection was significantly higher for both confirmed (29% increase) and confirmed or CIDT positive–only infections (91% increase). Incidence of confirmed *Cryptosporidium* infection was also significantly higher in 2016 compared with 2013–2015 (45% increase).

**TABLE 2:** Percentage change in incidence of confirmed and CIDT positive–only* bacterial and confirmed parasitic infections in 2016^†^ compared with 2013–2015 average annual incidence, by pathogen — FoodNet, 10 U.S. sites,^§^ 2013–2016

Pathogen	Confirmed			Confirmed or CIDT positive–only			
	2016 IR^¶^	% Change**	95% CI	2016 IR^¶^		% Change**	95% CI
*Campylobacter*	11.79	-11	-18 to -3	17.43		+3	-4 to +10
*Listeria* ^††^	0.26	+4	-18 to +30	—^§§^		—^§§^	—^§§^
*Salmonella*	15.40	+2	-4 to +8	16.66		+6	-1 to +12
*Shigella*	4.60	+7	-17 to +38	5.94		+25	-3 to +62
STEC^¶¶^	2.84	+21	+3 to +42	3.76		+43	+22 to +67
*Vibrio*	0.45	+2	-18 to +26	0.51		+16	-6 to +42
*Yersinia*	0.42	+29	+2 to +64	0.62		+91	+52 to +140
*Cryptosporidium*	3.70	+45	+11 to +89	—^§§^		—^§§^	—^§§^

**Abbreviations:** CI = confidence interval; CIDT = culture-independent diagnostic test; FoodNet = CDC’s Foodborne Diseases Active Surveillance Network; IR = incidence rate; STEC = Shiga toxin–producing *Escherichia coli*.

* CIDT positive only is defined as detection of the bacterial pathogen, or for STEC, Shiga toxin or the genes that encode a Shiga toxin, in a stool specimen or enrichment broth using a CIDT. Any positive CIDT result that was confirmed by culture is counted only among the confirmed infections. For STEC, only CIDT reports that were positive at a state public health laboratory were counted.

^†^ Data for 2016 are preliminary.

^§^ Connecticut, Georgia, Maryland, Minnesota, New Mexico, Oregon, Tennessee, and selected counties in California, Colorado, and New York.

^¶^ Per 100,000 population.

** Percentage change reported as increase (+) or decrease (-).

^††^ *Listeria* cases defined as isolation of *L. monocytogenes* from a normally sterile site, or in the setting of miscarriage or stillbirth, isolation of *L. monocytogenes* from placental or fetal tissue.

^§§^ All infections were confirmed.

^¶¶^ For STEC, all serogroups were combined, because it is not possible to distinguish between serogroups using CIDTs.

Among 7,554 confirmed *Salmonella* cases in 2016, serotype information was available for 6,583 (87%). The most common serotypes were Enteritidis (1,320; 17%), Newport (797; 11%), and Typhimurium (704; 9%). The incidence in 2016 compared with 2013–2015 was significantly lower for Typhimurium (18% decrease; CI = 7%–21%) and unchanged for Enteritidis and Newport. Among 208 (95%) speciated *Vibrio* isolates, 103 (50%) were *V. parahaemolyticus*, 35 (17%) were *V. alginolyticus*, and 26 (13%) were *V. vulnificus*. Among 1,394 confirmed and serogrouped STEC cases, 503 (36%) were STEC O157 and 891 (64%) were STEC non-O157. Among 586 (70%) STEC non-O157 isolates, the most common serogroups were O26 (190; 21%), O103 (178; 20%), and O111 (106; 12%). Compared with 2013–2015, the incidence of STEC non-O157 infections in 2016 was significantly higher (26% increase; CI = 9%–46%) and the incidence of STEC O157 was unchanged.

FoodNet identified 62 cases of postdiarrheal HUS in children aged <18 years (0.56 cases per 100,000) in 2015; 33 (56%) occurred in children aged <5 years (1.18 cases per 100,000). Compared with 2012–2014, in 2015, no significant differences in incidence among all children or children aged <5 years were observed.

## Discussion

The number of CIDT positive–only infections reported to FoodNet has been increasing markedly since 2013, as more clinical laboratories adopt CIDTs. Initially, increases were primarily limited to *Campylobacter* and STEC; followed by substantial increases in *Salmonella* and *Shigella* beginning in 2015 (*6*). The pattern continued in 2016, with large increases in the number of CIDT positive–only *Vibrio* and *Yersinia* infections. When including both confirmed and CIDT positive–only infections, incidence rates in 2016 were higher for each of these six pathogens. The increasing use of CIDTs presents challenges when interpreting the corresponding increases in incidence. For example, the incidence of confirmed *Campylobacter* infections in 2016 was significantly lower than the 2013–2015 average. However, when including CIDT positive–only infections, a slight but not significant increase occurred. For STEC and *Yersinia*, the incidence of confirmed infections alone and confirmed or CIDT positive–only infections in 2016 were both significantly higher than the 2013–2015 average; the magnitude of change approximately doubled when analyzing CIDT positive–only infections.

Because of the ease and increasing availability of CIDTs, testing for some pathogens might be increasing as health care provider behaviors and laboratory practices evolve (*2*). Among clinical laboratories in the FoodNet catchment, the use of CIDTs to detect *Salmonella*, for which the only CIDTs available are DNA-based gastrointestinal syndrome panels, increased from 2 per 460 laboratories (<1%) in 2013 to 59 per 421 laboratories (14%) in 2016 (FoodNet, unpublished data). This increased use paralleled significant increases in incidence of *Cryptosporidium*, STEC, and *Yersinia*, and slight but not significant increases in incidence of *Campylobacter, Salmonella, Shigella,* and *Vibrio*, all of which are also included in these panel tests. The increase in STEC incidence is driven by the increase in STEC non-O157, which is not typically included in routine stool culture testing because it requires specialized methods. Routine stool cultures performed in clinical laboratories typically include methods that identify only *Salmonella*, *Campylobacter*, *Shigella*, and for some laboratories, STEC O157 (*4*,*5*). The increased use of the syndrome panel tests might increase identification, and thus, improve incidence estimates of pathogens for which testing was previously limited.

Results are more quickly obtained using CIDTs than traditional culture methods (*3*). Because of this, health care providers might be more likely to order a CIDT than traditional culture (*2*). Increased testing might identify infections that previously would have remained undiagnosed. However, sensitivity and specificity vary by test type. Evaluations of DNA-based syndrome panel tests have indicated high sensitivity and specificity for most targets (*3*). However, among pathogens for which antigen-based CIDTs are often used, such as *Campylobacter* and *Cryptosporidium*, sensitivity and specificity have varied more widely, with a large number of false positive results (*7*,*8*). Including CIDT positive infections to calculate incidence, some of which could be false positives, might provide an inaccurate estimate. When interpreting incidence and trends in light of changing diagnostic testing, considering frequency of testing, sensitivity, and specificity of these tests is important. The observed increases in incidence of confirmed or CIDT positive–only infections in 2016 compared with 2013–2015 could be caused by increased testing, varying test sensitivity, an actual increase in infections, or a combination of these reasons.

These changes in testing are also important to consider when monitoring progress toward *Healthy People 2020* objectives.^††^ The current objectives were created before the use of CIDTs and were based on confirmed infections. In the future, just as incidence measures should adjust for these changes, objectives should also be evaluated in light of changing diagnostics.

CIDTs pose additional challenges because they do not yield the bacterial isolates necessary for essential public health surveillance activities, such as monitoring trends in pathogen subtypes, conducting molecular testing, detecting outbreaks and implicating vehicles, and determining antimicrobial susceptibility. Reflex culture performed to yield an isolate places an additional burden on laboratories’ budgets, personnel, and time. Specimen submission requirements differ by state and pathogen, and this responsibility often falls to state public health laboratories (*9*). As CIDT use increases and more pathogens are affected, state public health laboratories will be challenged to sufficiently increase their testing capacity and will likely have to prioritize specimens on which to perform reflex culture (*10*). Clinical laboratories should review state specimen submission requirements and the Association of Public Health Laboratories guidelines^§§^ for reflex culture and submission of CIDT positive specimens.

The findings in this report are subject to at least two limitations. First, the changing diagnostic landscape with unknown changes in frequency of testing, varying test performance, and decreasing availability of isolates for subtyping make interpreting incidence and trends more difficult. Second, changes in health care–seeking behavior, access to health services, or other population characteristics might have changed since the comparison period, which could affect incidence.

Foodborne illness remains a substantial public health concern in the United States. Previous analyses have indicated that the number of infections far exceeds those diagnosed; CIDTs might be making those infections more visible (*11*). Most foodborne infections can be prevented, and substantial progress has been made in the past in decreasing contamination of some foods and reducing illness caused by some pathogens. More prevention measures are needed. Surveillance data can provide information on where to target these measures. However, to accurately interpret FoodNet surveillance data in light of changes in diagnostic testing, more data and analytic tools are needed to adjust for changes in testing practices and differences in test characteristics. FoodNet is collecting more data and developing those tools. With these, FoodNet will continue to track the needed progress toward reducing foodborne illness.

**Summary**

**What is already known about this topic?**

The incidence of infections transmitted commonly through food has remained largely unchanged for many years. Culture-independent diagnostic tests (CIDTs) are increasingly used by clinical laboratories to detect enteric infections.

**What is added by this report?**

Compared with the 2013–2015 average annual incidence, the 2016 incidence of confirmed *Campylobacter* infections was lower, incidences of confirmed Shiga toxin-producing *Escherichia coli* (STEC), *Yersinia*, and *Cryptosporidium* infections were higher, and incidences of confirmed or CIDT positive–only STEC and *Yersinia* infections were higher. However, CIDTs complicate the interpretation of surveillance data; testing for pathogens might occur more frequently because of changes in either health care provider behaviors or laboratory testing practices. A large proportion of CIDT positive specimens were not reflex cultured, which is necessary to obtain isolates for distinguishing pathogen subtypes, determining antimicrobial resistance, monitoring trends, and detecting outbreaks.

**What are the implications for public health practice?**

Some information about the bacteria causing infections, such as subtype and antimicrobial susceptibility, can only be obtained for CIDT positive specimens if reflex culture is performed. Increasing use of CIDTs affects the interpretation of public health surveillance data and ability to monitor progress toward prevention measures.

## Footnotes

* Connecticut, Georgia, Maryland, Minnesota, New Mexico, Oregon, Tennessee, and selected counties in California, Colorado, and New York (https://www.cdc.gov/foodnet).

† Culturing of a specimen with a positive culture-independent diagnostic test (CIDT) result.

§ For Shiga toxin-producing *Escherichia coli*, only CIDT reports that were positive at a state public health laboratory were counted.

¶ Excludes Shiga toxin–positive reports from clinical laboratories that were Shiga toxin–negative at a public health laboratory (n=568).

†† https://www.healthypeople.gov/2020/topics-objectives/topic/food-safety/objectives.

§§ https://www.aphl.org/aboutAPHL/publications/Documents/FS-Enteric_Pathogens_Guidelines_0216.pdf.
