# *Erratum:* Vol. 66, No. 21

**DOI:** 10.15585/mmwr.mm6624a6

**Published:** 2017-06-23

In “Notifiable Diseases and Mortality Tables,” on pages ND403–22, weekly case counts were inadvertently omitted from the tables “Provisional cases of selected* infrequently reported notifiable diseases (<1,000 cases reported during the preceding year) — United States, week ending May 27, 2017 (21st week)^†^” and “Provisional cases of selected notifiable diseases (≥1,000 cases reported during the preceding year), and selected* low frequency diseases, United States and U.S. territories, weeks ending May 27, 2017, and May 28, 2016 (21st week).^†^” The updated Week 21 tables can be found at https://wonder.cdc.gov/ and at https://data.cdc.gov/.

